# Supplementary material for: Enhancer architecture sensitizes cell specific responses to Notch gene dose via a bind and discard mechanism
Source: eLife. 2020 Apr 16;9:e53659. doi: 10.7554/eLife.53659 (PMC7213981; doi:10.7554/eLife.53659)
Supplement: Supplementary file 1. — All genotypes of Drosophila fly lines mated to generate the offspring and data shown in each experimental Figure. [file elife-53659-supp1.docx]

Supplementary File 1. Genetic crosses performed to generate the analyzed progeny.

|  | Female | Male |
| --- | --- | --- |
| Figure 1G | *6SG-lacZ_86Fb_/6SG-lacZ_86Fb_* | *yw^1118^* |
| Figure 1H | *6SG-lacZ_86Fb_/6SG-lacZ_86Fb_* | *6SG-lacZ_86Fb_/6SG-lacZ_86Fb_* |
| Figure 1I | *6SG-lacZ_51c_/6SG-lacZ_51c_;6SG-lacZ_86Fb_/6SG-lacZ_86Fb_* | *6SG-lacZ_51c_/6SG-lacZ_51c_;6SG-lacZ_86Fb_/6SG-lacZ_86Fb_* |
| Figure 1J | *12CG-lacZ_51c_/12CG-lacZ_51c_;12CG-lacZ_86Fb_/12CG-lacZ_86Fb_* | *12CG-lacZ_51c_/12CG-lacZ_51c_;12CG-lacZ_86Fb_/12CG-lacZ_86Fb_* |
| Figure 1K | *G2S-lacZ_86Fb_/G2S-lacZ_86Fb_* | *G2S-lacZ_86Fb_/G2S-lacZ_86Fb_* |
|  | *G6S-lacZ_86Fb_/G6S-lacZ_86Fb_* | *G6S-lacZ_86Fb_/G6S-lacZ_86Fb_* |
|  | *G24S-lacZ_86Fb_/G24S-lacZ_86Fb_* | *G24S-lacZ_86Fb_/G24S-lacZ_86Fb_* |
| Figure 1L | *6SG-lacZ_86Fb_/6SG-lacZ_86Fb_* | *6SG-lacZ_86Fb_/6SG-lacZ_86Fb_* |
|  | *12CG-lacZ_86Fb_/12CG-lacZ_86Fb_* | *12CG-lacZ_86Fb_/12CG-lacZ_86Fb_* |
| Figure 2A | *N^55e11^* *FRT19A/FM7c* | *yw^1118^* |
| Figure 2B | *N^55e11^* *FRT19A/FM7c* | *G6S-lacZ_86Fb_/G6S-lacZ_86Fb_* |
| Figure 2C | *N^55e11^* *FRT19A/FM7c* | *yw^1118^* |
|  | *N^55e11^* *FRT19A/FM7c* | *6SG-lacZ_86Fb_/6SG-lacZ_86Fb_* |
|  | *yw^1118^* | *6SG-lacZ_86Fb_/6SG-lacZ_86Fb_* |
| Figure 2D | *PBac{N-GFP.FLAG}/PBac{N-GFP.FLAG}* | *PBac{N-GFP.FLAG}/PBac{N-GFP.FLAG}* |
| Figure 2E | *6SG-lacZ_51c_/6SG-lacZ_51c_;PBac{N-GFP.FLAG}/PBac{N-GFP.FLAG}* | *6SG-lacZ_51c_/6SG-lacZ_51c_;PBac{N-GFP.FLAG}/PBac{N-GFP.FLAG}* |
| Figure 2F | *6SG-lacZ_51c_/6SG-lacZ_51c_* | *6SG-lacZ_51c_/6SG-lacZ_51c_* |
|  | *6SG-lacZ_51c_/6SG-lacZ_51c_;PBac{N-GFP.FLAG}/PBac{N-GFP.FLAG}* | *6SG-lacZ_51c_/6SG-lacZ_51c_;PBac{N-GFP.FLAG}/PBac{N-GFP.FLAG}* |
|  | *6SG-lacZ_51c_/6SG-lacZ_51c_;6SG-lacZ_86Fb_/6SG-lacZ_86Fb_* | *H^1^/TM6B, Tb^1^* |
| Figure 2G | *yw^1118^* | *H^1^/TM6B, Tb^1^* |
| Figure 2H | *6SG-lacZ_51c_/6SG-lacZ_51c_;6SG-lacZ_86Fb_/6SG-lacZ_86Fb_* | *H^1^/TM6B, Tb^1^* |
| Figure 2I | *yw^1118^* | *H^1^/TM6B, Tb^1^* |
|  | *6SG-lacZ_51c_/6SG-lacZ_51c_;6SG-lacZ_86Fb_/6SG-lacZ_86Fb_* | *H^1^/TM6B, Tb^1^* |
|  | *12CG-lacZ_51c_/12CG-lacZ_51c_;12CG-lacZ_86Fb_/12CG-lacZ_86Fb_* | *H^1^/TM6B, Tb^1^* |
| Figure 2J | *N^55e11^* *FRT19A/FM7c* | *yw^1118^* |
| Figure 2K | *G6S-lacZ_86Fb_/G6S-lacZ_86Fb_* | *G6S-lacZ_86Fb_/G6S-lacZ_86Fb_* |
| Figure 2L | *N^55e11^* *FRT19A/FM7c* | *yw^1118^* |
|  | *N^55e11^* *FRT19A/FM7c* | *G6S-lacZ_86Fb_/G6S-lacZ_86Fb_* |
|  | *6SG-lacZ_51c_/6SG-lacZ_51c_;6SG-lacZ_86Fb_/6SG-lacZ_86Fb_* | *yw^1118^* |
|  | *yw^1118^* | *H^1^/TM6B, Tb^1^* |
|  | *6SG-lacZ_51c_/6SG-lacZ_51c_;6SG-lacZ_86Fb_/6SG-lacZ_86Fb_* | *H^1^/TM6B, Tb^1^* |
| Figure 3A | *6SG-lacZ_51c_/6SG-lacZ_51c_;6SG-lacZ_86Fb_/6SG-lacZ_86Fb_* | *yw^1118^* |
|  | *6SG-lacZ_51c_/6SG-lacZ_51c_;6SG-lacZ_86Fb_/6SG-lacZ_86Fb_* | *FRT80 cdk8^K185^/TM6B* |
|  | *6SG-lacZ_51c_/6SG-lacZ_51c_;6SG-lacZ_86Fb_/6SG-lacZ_86Fb_* | *FRT82 cycC^Y5^/TM6B* |
|  | *6SG-lacZ_51c_/6SG-lacZ_51c_;6SG-lacZ_86Fb_/6SG-lacZ_86Fb_* | *kto^T241^ FRT80B/TM6B, Tb^1^* |
|  | *6SG-lacZ_51c_/6SG-lacZ_51c_;6SG-lacZ_86Fb_/6SG-lacZ_86Fb_* | *skd^T13^ FRT80B/TM6B, Tb^1^* |
|  | *6SG-lacZ_51c_/6SG-lacZ_51c_;6SG-lacZ_86Fb_/6SG-lacZ_86Fb_* | *ago^1^ FRT80B/TM6B, Tb^1^* |
| Figure 3B | *N^55e11^* *FRT19A/FM7c* | *yw^1118^* |
|  | *N^55e11^* *FRT19A/FM7c* | *FRT80 cdk8^K185^/TM6B* |
|  | *N^55e11^* *FRT19A/FM7c* | *FRT82 cycC^Y5^/TM6B* |
|  | *N^55e11^* *FRT19A/FM7c* | *kto^T241^ FRT80B/TM6B, Tb^1^* |
|  | *N^55e11^* *FRT19A/FM7c* | *skd^T13^ FRT80B/TM6B, Tb^1^* |
|  | *N^55e11^* *FRT19A/FM7c* | *ago^1^ FRT80B/TM6B, Tb^1^* |
| Figure 3D | *G24S-GFP_51c_/G24S-GFP_51c_;G24S-GFP_86Fb_/G24S-GFP_86Fb_* | *yw^1118^* |
|  | *G24S_51c_/G24S_51c_; G24S_86Fb_/G24S_86Fb_* | *yw^1118^* |
|  | *G24S-GFP_51c_/G24S-GFP_51c_;G24S-GFP_86Fb_/G24S-GFP_86Fb_* | *skd^T413^ FRT80B/TM6B, Tb^1^* |
|  | *G24S_51c_/G24S_51c_; G24S_86Fb_/G24S_86Fb_* | *skd^T413^ FRT80B/TM6B, Tb^1^* |
| Figure 4C | *6S-GFP_22A_/6S-GFP_22A_;G6S-lacZ_86Fb_/G6S-lacZ_86Fb_* | *yw^1118^* |
|  | *6S-GFP_22A_/6S-GFP_22A_;G6S-lacZ_86Fb_/G6S-lacZ_86Fb_* | *skd^T413^ FRT80B/TM6B, Tb^1^* |
|  | *6S-GFP_22A_/6S-GFP_22A_;(G6S)2-lacZ_86Fb_/(G6S)2-lacZ_86Fb_* | *yw^1118^* |
|  | *6S-GFP_22A_/6S-GFP_22A_;(G6S)2-lacZ_86Fb_/(G6S)2-lacZ_86Fb_* | *skd^T413^ FRT80B/TM6B, Tb^1^* |
|  | *6S-GFP_22A_/6S-GFP_22A_;(G6S)3-lacZ_86Fb_/(G6S)3-lacZ_86Fb_* | *yw^1118^* |
|  | *6S-GFP_22A_/6S-GFP_22A_;(G6S)3-lacZ_86Fb_/(G6S)3-lacZ_86Fb_* | *skd^T413^ FRT80B/TM6B, Tb^1^* |
| Figure 4D | *6SG-lacZ_51c_/6SG-lacZ_51c_;G-lacZ_86Fb_/G-lacZ_86Fb_* | *yw^1118^* |
| Figure 4E | *6S-GFP_22A_/6S-GFP_22A_;G6S-lacZ_86Fb_/G6S-lacZ_86Fb_* | *yw^1118^* |
| Figure 4F | *6S-GFP_22A_/6S-GFP_22A_;(G6S)2-lacZ_86Fb_/(G6S)2-lacZ_86Fb_* | *yw^1118^* |
| Figure 4G | *6S-GFP_22A_/6S-GFP_22A_;(G6S)3-lacZ_86Fb_/(G6S)3-lacZ_86Fb_* | *yw^1118^* |
| Figure 4J | *N^55e11^* *FRT19A/FM7c* | *yw^1118^* |
|  | *N^55e11^* *FRT19A/FM7c* | *FRT80 cdk8^K185^/TM6B* |
|  | *N^55e11^* *FRT19A/FM7c* | *FRT82 cycC^Y5^/TM6B* |
|  | *N^55e11^* *FRT19A/FM7c* | *kto^T241^ FRT80B/TM6B, Tb^1^* |
|  | *N^55e11^* *FRT19A/FM7c* | *skd^T13^ FRT80B/TM6B, Tb^1^* |
|  | *H^1^/TM6B, Tb^1^* | *yw^1118^* |
|  | *H^1^/TM6B, Tb^1^* | *FRT80 cdk8^K185^/TM6B* |
|  | *H^1^/TM6B, Tb^1^* | *FRT82 cycC^Y5^/TM6B* |
|  | *H^1^/TM6B, Tb^1^* | *kto^T241^ FRT80B/TM6B, Tb^1^* |
|  | *H^1^/TM6B, Tb^1^* | *skd^T13^ FRT80B/TM6B, Tb^1^* |
| Figure 5B | *G6S-lacZ_86Fb_/G6S-lacZ_86Fb_* | *G6S-lacZ_86Fb_/G6S-lacZ_86Fb_* |
|  | *G6Sm8-lacZ_86Fb_/G6Sm8-lacZ_86Fb_* | *G6Sm8-lacZ_86Fb_/G6Sm8-lacZ_86Fb_* |
| Figure 5C | *Zld-lacZ_86Fb_ /Zld-lacZ_86Fb_* | *Zld-lacZ_86Fb_ /Zld-lacZ_86Fb_* |
|  | *Zld6S-lacZ_86Fb_ /Zld6S-lacZ_86Fb_* | *Zld6S-lacZ_86Fb_ /Zld6S-lacZ_86Fb_* |
|  | *Zld12C-lacZ_86Fb_ /Zld12C-lacZ_86Fb_* | *Zld12C-lacZ_86Fb_ /Zld12C-lacZ_86Fb_* |
| Figure 5D | *Ebox-lacZ_86Fb_/Ebox-lacZ_86F_* | *Ebox-lacZ_86Fb_/Ebox-lacZ_86F_* |
|  | *Ebox6S-lacZ_86Fb_/Ebox6S-lacZ_86Fb_* | *Ebox6S-lacZ_86Fb_/Ebox6S-lacZ_86Fb_* |
|  | *Ebox12C-lacZ_86Fb_/Ebox12C-lacZ_86Fb_* | *Ebox12C-lacZ_86Fb_/Ebox12C-lacZ_86Fb_* |
| Figure 5E | *N^55e11^* *FRT19A/FM7c* | *0S-lacZ_86Fb_/0S-lacZ_86Fb_* |
|  | *N^55e11^* *FRT19A/FM7c* | *6S-lacZ_86Fb_/6S-lacZ_86Fb_* |
| Figure 1 —figure supplement 2A | *G-lacZ_86Fb_/G-lacZ_86Fb_* | *G-lacZ_86Fb_/G-lacZ_86Fb_* |
| Figure 1 —figure supplement 2B | *6S-lacZ_86Fb_/6S-lacZ_86Fb_* | *6S-lacZ_86Fb_/6S-lacZ_86Fb_* |
| Figure 1 —figure supplement 2C | *G-lacZ_86Fb_/G-lacZ_86Fb_* | *G-lacZ_86Fb_/G-lacZ_86Fb_* |
|  | *6S-lacZ_86Fb_/6S-lacZ_86Fb_* | *6S-lacZ_86Fb_/6S-lacZ_86Fb_* |
|  | *6SmutG-lacZ_86Fb_/6SmutG-lacZ_86Fb_* | *6SmutG-lacZ_86Fb_/6SmutG-lacZ_86Fb_* |
| Figure 1 —figure supplement 2D | *6SG-lacZ_51c_/6SG-lacZ_51c_* | *6SG-lacZ_51c_/6SG-lacZ_51c_* |
| Figure 1 —figure supplement 2E | *6SG-lacZ_86Fb_/6SG-lacZ_86Fb_* | *6SG-lacZ_86Fb_/6SG-lacZ_86Fb_* |
| Figure 1 —figure supplement 2F | *G6S-lacZ_86Fb_/G6S-lacZ_86Fb_* | *G6S-lacZ_86Fb_/G6S-lacZ_86Fb_* |
| Figure 1 —figure supplement 2G | *6SG-lacZ_51c_/6SG-lacZ_51c_;6SG-lacZ_86Fb_/6SG-lacZ_86Fb_* | *yw^1118^* |
| Figure 1 —figure supplement 2H | *6SG-lacZ_51c_/6SG-lacZ_51c_* | *6SG-lacZ_51c_/6SG-lacZ_51c_* |
|  | *6SG-lacZ_86Fb_/6SG-lacZ_86Fb_* | *6SG-lacZ_86Fb_/6SG-lacZ_86Fb_* |
|  | *G6S-lacZ_86Fb_/G6S-lacZ_86Fb_* | *G6S-lacZ_86Fb_/G6S-lacZ_86Fb_* |
|  | *6SG-lacZ_51c_/6SG-lacZ_51c_;6SG-lacZ_86Fb_/6SG-lacZ_86Fb_* | *yw^1118^* |
| Figure 2 —figure supplement 1 | *N^1^/FM7c* | *6SG-lacZ_86Fb_/6SG-lacZ_86Fb_* |
|  | *N^1^/FM7c* | *yw^1118^* |
| Figure 3 —figure supplement 1A | *6SG-lacZ_51c_/6SG-lacZ_51c_;6SG-lacZ_86Fb_/6SG-lacZ_86Fb_* | *yw^1118^* |
|  | *6SG-lacZ_51c_/6SG-lacZ_51c_;6SG-lacZ_86Fb_/6SG-lacZ_86Fb_* | *kto^T631^ FRT80B/TM6B, Tb^1^* |
|  | *6SG-lacZ_51c_/6SG-lacZ_51c_;6SG-lacZ_86Fb_/6SG-lacZ_86Fb_* | *skd^T413^ FRT80B/TM6B, Tb^1^* |
|  | *6SG-lacZ_51c_/6SG-lacZ_51c_;6SG-lacZ_86Fb_/6SG-lacZ_86Fb_* | *ago^3^ FRT80B/TM6B, Tb^1^* |
| Figure 3 —figure supplement 1B | *N^55e11^* *FRT19A/FM7c* | *yw^1118^* |
|  | *N^55e11^* *FRT19A/FM7c* | *kto^T631^ FRT80B/TM6B, Tb^1^* |
|  | *N^55e11^* *FRT19A/FM7c* | *skd^T413^ FRT80B/TM6B, Tb^1^* |
|  | *N^55e11^* *FRT19A/FM7c* | *ago^3^ FRT80B/TM6B, Tb^1^* |
| Figure 4 —figure supplement 1A | *yw^1118^* | *6S-GFP_86Fb_/6S-GFP_86Fb_* |
|  | *N^55e11^* *FRT19A/FM7i, ActGFP* | *6S-GFP_86Fb_/6S-GFP_86Fb_* |
